# Eradication of *Acinetobacter baumannii*/*Enterobacter cloacae* complex in an open proximal tibial fracture and closed drop foot correction with a multidisciplinary approach using the Taylor Spatial Frame^®^: a case report

**DOI:** 10.1186/s40001-019-0360-2

**Published:** 2019-01-19

**Authors:** G. Siebenbürger, B. Grabein, T. Schenck, C. Kammerlander, W. Böcker, C. Zeckey

**Affiliations:** 1 Department for General, Trauma and Reconstructive Surgery, University Hospital, LMU Munich, Ludwig-Maximilians-Universität München, Marchioninistr. 15, 81377 München, Germany; 2 Department for Clinical Microbiology and Hospital Hygiene, University Hospital, LMU Munich, Ludwig-Maximilians-Universität München, Marchioninistr. 15, 81377 München, Germany; 3 Department for Hand-, Plastic and Aesthetic Surgery, University Hospital, LMU Munich, Ludwig-Maximilians-Universität München, Nussbaumstr. 20, 80336 München, Germany

**Keywords:** Proximal tibial fracture, Locked plating, Open fracture, *Acinetobacter baumannii*, Complication, Outcome, Hexapod external fixation, Latissimus dorsi muscle flap, Antibiotics, Infection

## Abstract

**Background:**

Multi-drug-resistant bacteria (e.g. Carbapenem-resistant *Acinetobacter baumannii*, extended-spectrum betalactamase or carbapenemase-producing enterobacteriaceae) are emerging in early-onset infections. So far, there is no report describing the eradication of these bacteria in a osseous infection of an open proximal tibial fracture in combination with the hexapod technology to address both osseous consolidation and closed drop foot correction.

**Case presentation:**

After sustaining a proximal tibial fracture (Gustilo 3B), a 41-year-old man was primarily treated with open reduction and internal fixation by a locking plate and split-thickness skin graft in the home country. At the time of admission to our hospital there was a significant anterolateral soft tissue defect covered with an already-necrotic split-thickness graft and suspicious secretion. CAT and MRI scans revealed no signs of osseous healing, intramedullary distinctive osteomyelitis, as well as a large abscess zone in the dorsal compartment. Multiple wound smears showed multi-drug-resistant bacteria: *Acinetobacter baumannii* (Carbapenem resistant) as well as *Enterobacter cloacae* complex (AmpC overexpression). After implant removal, excessive osseous and intramedullary debridements using the Reamer Irrigator Aspirator (RIA^®^) as well as initial negative pressure wound therapy were performed. Colistin hand-modelled chains and sticks were applied topically as well as an adjusted systemic antibiotic scheme was applied. After repetitive surgical interventions, the smears showed bacterial eradication and the patient underwent soft tissue reconstruction with a free vascularized latissimus dorsi muscle flap. External fixation was converted to a hexapod fixator (TSF^®^) to correct primary varus displacement, axial assignment and secure osseous healing. A second ring was mounted to address the fixed drop foot in a closed fashion without further intervention. At final follow-up, 12 months after trauma, the patient showed good functional recovery with osseous healing, intact soft tissue with satisfactory cosmetics and no signs of reinfection.

**Conclusions:**

A multidisciplinary approach with orthopaedic surgeons for debridement, planning and establishing osseous and joint correction and consolidation, plastic surgeons for microvascular muscle flaps for soft tissue defect coverage as well as clinical microbiologists for the optimized anti-infective treatment is essential in these challenging rare cases.

**Level of evidence:**

Level IV.

## Background

Open tibial fractures are common in motorcycle accidents due to high-energy trauma and mechanical exposition of the lower limb [1, 2]. The initial surgical treatment in open fractures involves proper debridement followed by external fixation, nailing or open reduction and internal fixation depending on soft tissue status and condition of the patient [3–5]. Major complications include osseous and soft tissue damage and necrosis, infection and in complex injury patterns loss of the lower limb [6]. Multi-drug-resistant bacteria (e.g. Carbapenem-resistant *Acinetobacter baumannii*, extended-spectrum betalactamase or carbapenemase-producing enterobacteriaceae) are emerging in early-onset infections especially, i.e. in contaminated war injuries and present a profound problem for eradication of the infection [7–12]. Literature revealed a very limited amount of reports of eradication in case of infection, mainly in case of total knee or total hip arthroplasty. So far, there is no report describing the eradication in a osseous infection in combination with the hexapod technology to address both, osseous consolidation and closed drop foot correction.

We present a case of an open proximal tibial fracture with early-onset infection that was treated in a multidisciplinary concept together with clinical microbiologists to handle the severe soft tissue and osseous infection with multi-drug-resistant bacteria as well as plastic surgeons to encounter the distinctive soft tissue defect zone. Aggressive surgical debridement and local antibiotics in high dose formed the fundament of this successful case management. Complex external fixation and osseous as well as joint correction with a hexapod fixator in combination with an individualized systemic antibiotic therapy and demanding soft tissue reconstruction with a free muscle flap led to osseous healing with good functional and cosmetic outcome 1 year after surgery.

## Case presentation

**Fig. 1:**
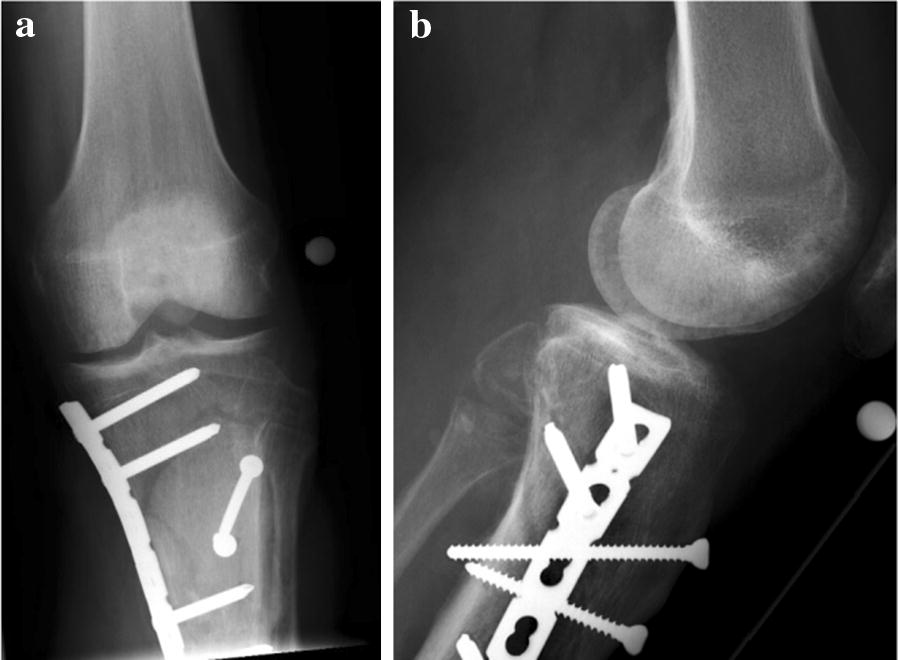
X-rays at first admission (AO type 41-A2, Anderson Gustilo IIIb, Tscherne/Oestern type III open fracture) after ORIF in Bulgaria. **a** X-ray a.p. view and **b** X-ray lateral view

**Fig. 2:**
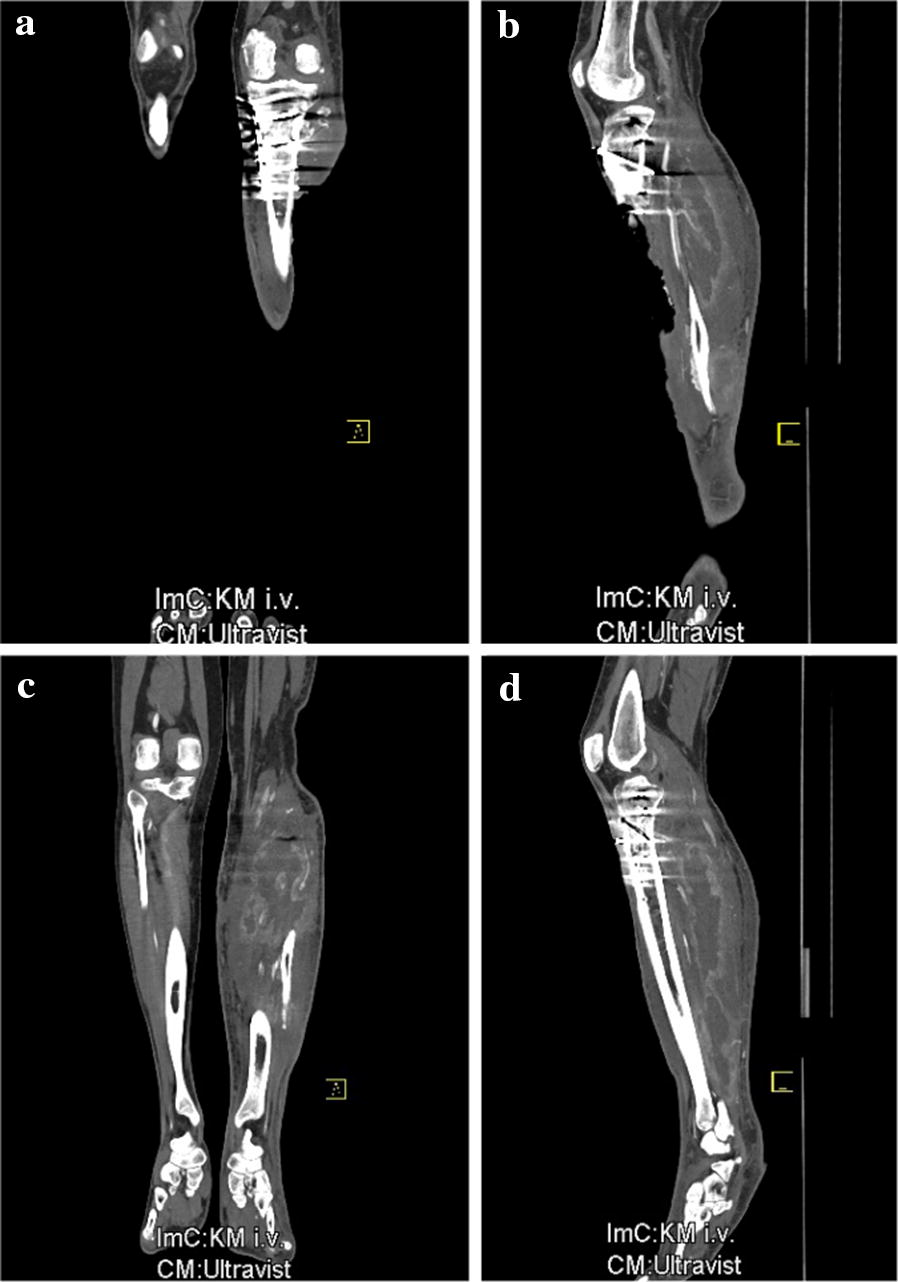
CAT scan at first admission showing severe soft tissue defect zone and abscess formation in the dorsal lower limb muscles. **a** CT coronary plane showing large lateral defect zone, **b** sagittal plane with large lateroventral defect zone, **c** CT coronary plane showing large abscess in the dorsal soft tissue and **d** sagittal plane showing large dorsal abscess zone

**Fig. 3:**
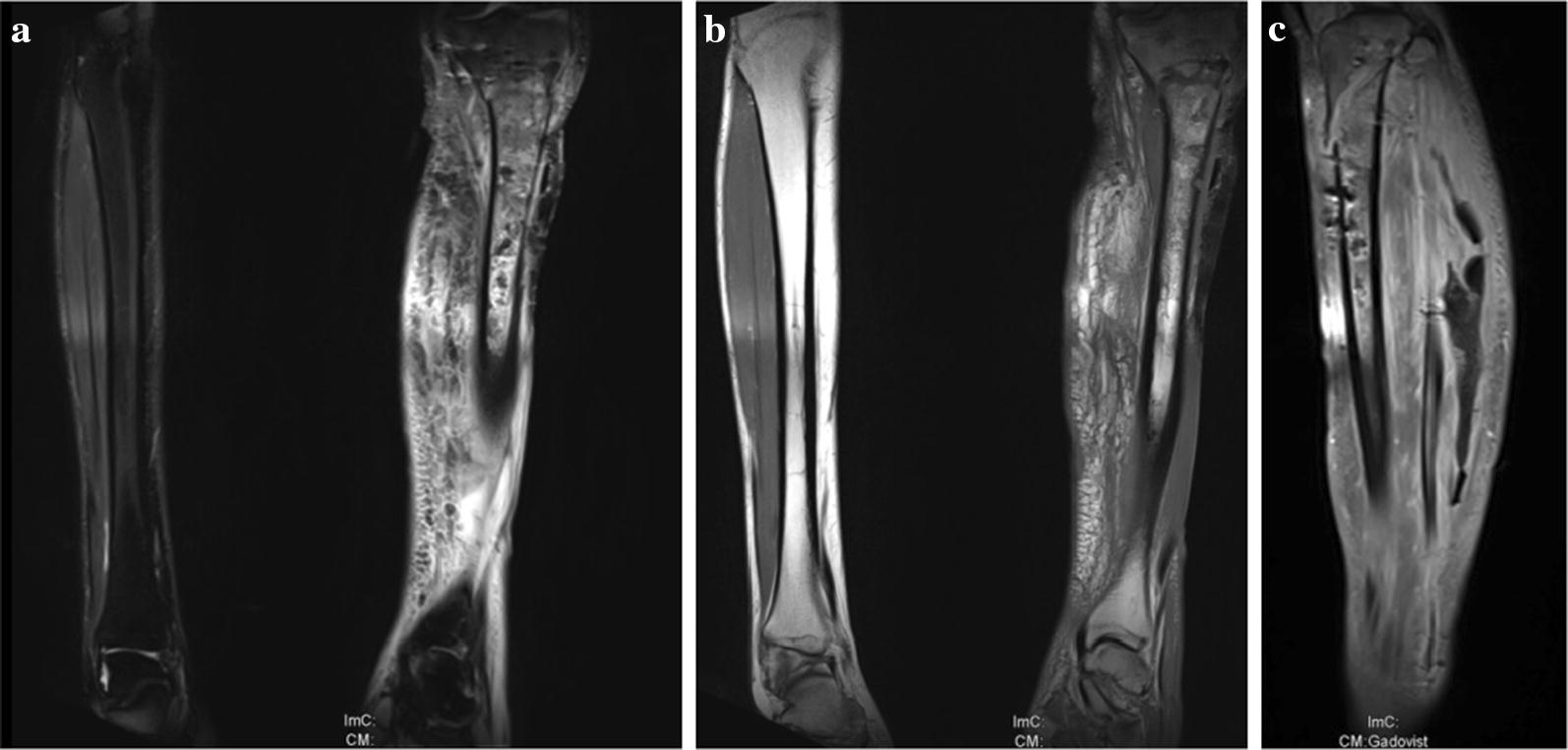
MRI scan at first admission showing septic pseudarthrosis, osteomyelitis and soft tissue abscess STIR/T1. **a** MRI coronary plane STIR sequence, **b** MRI coronary plane T1 sequence and **c** MRI sagittal plane STIR sequence

**Fig. 4:**
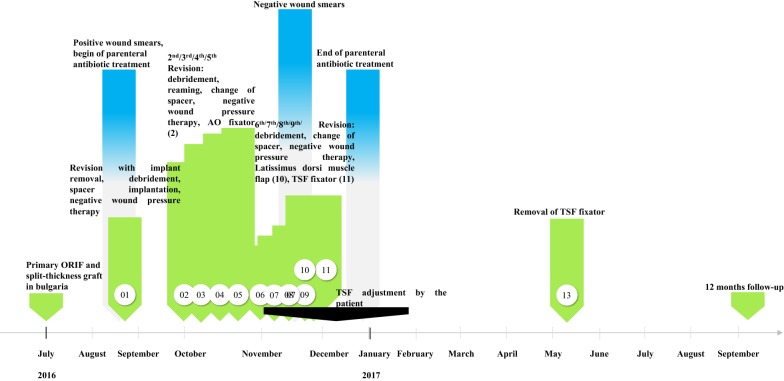
Timeline of the complete clinical course

After sustaining a motorcycle accident in July 2016, a 41-year-old man was initially treated with open reduction and internal fixation with a locking plate and single compression screw (LCP plate^®^, DePuy Synthes GmbH, Zuchwil, Switzerland) together with a split-thickness skin graft in Bulgaria (Fig. 1). The fracture pattern showed a proximal tibial fracture (AO/OTA type 41-A2, Anderson Gustilo IIIb, Tscherne/Oestern type III open fracture) and a proximal fibula fracture with consecutive sensomotoric lesion of the peroneal nerve [13–17]. After admission to our hospital in September 2016, initial diagnostics including plain radiographs and a pan CT/MRI scan revealed an onset of septic pseudarthrosis in the proximal tibia, intramedullary osteomyelitis, a large abscess zone with contrast agent capturing 30 cm in the dorsal compartment and necrotic avascular muscle areas, a loss of the anterior tibial artery as well as a large anterolateral soft tissue defect covered with necrotic split-thickness graft that was transplanted onto bone and osteosynthesis material at the primary hospital in Bulgaria (Figs. 2, 3). Mobilization was painful due to the clinically unstable osseous situation and reduced accordingly. Laboratory chemical infection parameters were slightly increased (CRP 2.7 mg/dl norm value < 0.5; leukocytes 6.9 G/l norm value 3.90–9.80). Local wound smears showed *Acinetobacter baumannii* (Carbapenem resistant) as well as *Enterobacter cloacae* complex (overexpression of AmpC-Betalactamase and fluoroquinolone resistant) in the large anterolateral defect zone and intramedullary in the proximal tibia to the distal third of the tibial shaft. For the complete clinical course, see the timeline (Fig. 4).

### Orthopaedic surgical procedure

**Fig. 5:**
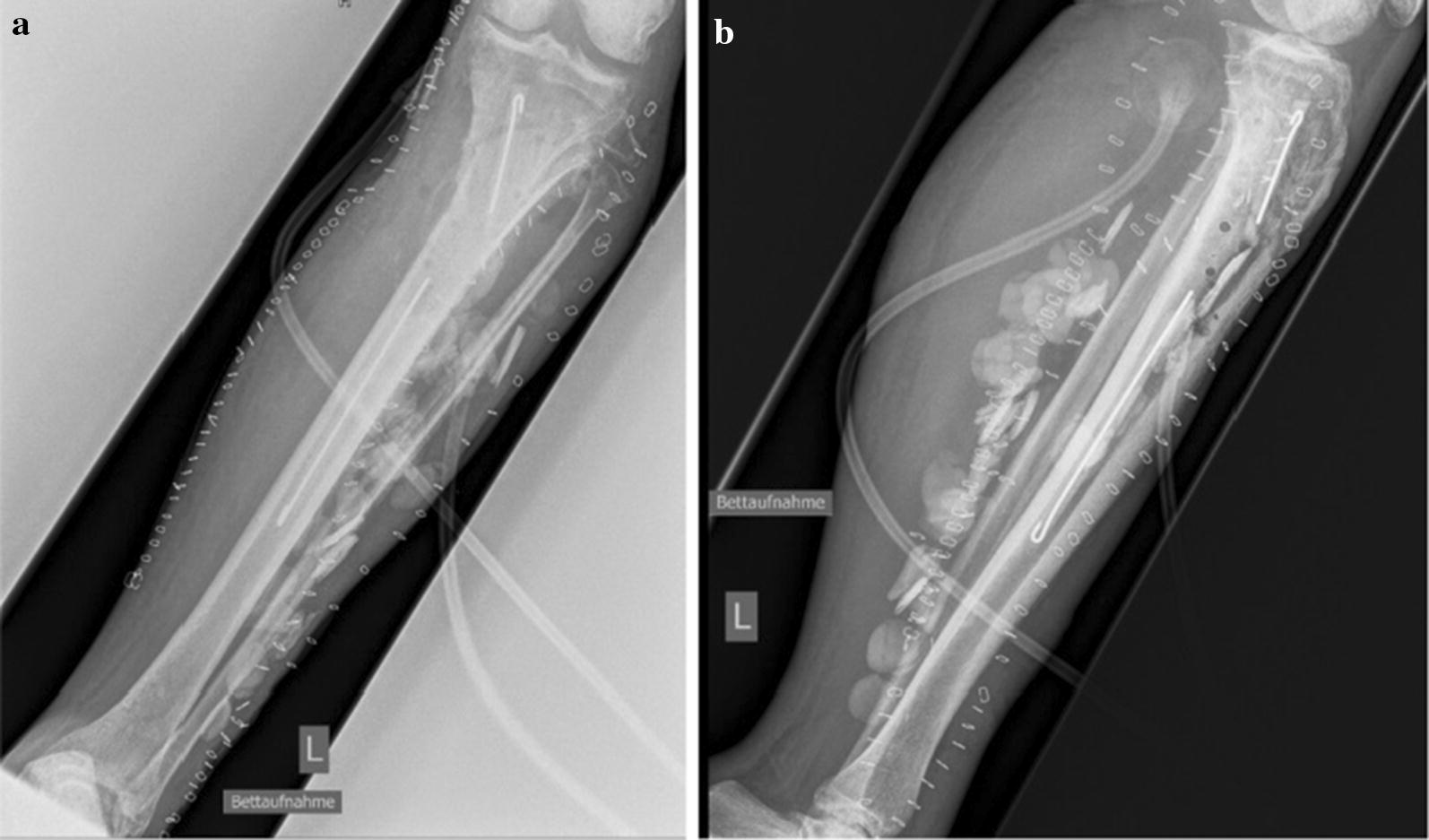
Plain X-rays showing reamed intramedullary zone, implanted spacer (sticks and chains) as well as negative pressure wound therapy in situ. **a** X-ray a.p. view and **b** X-ray lateral view

**Fig. 6:**
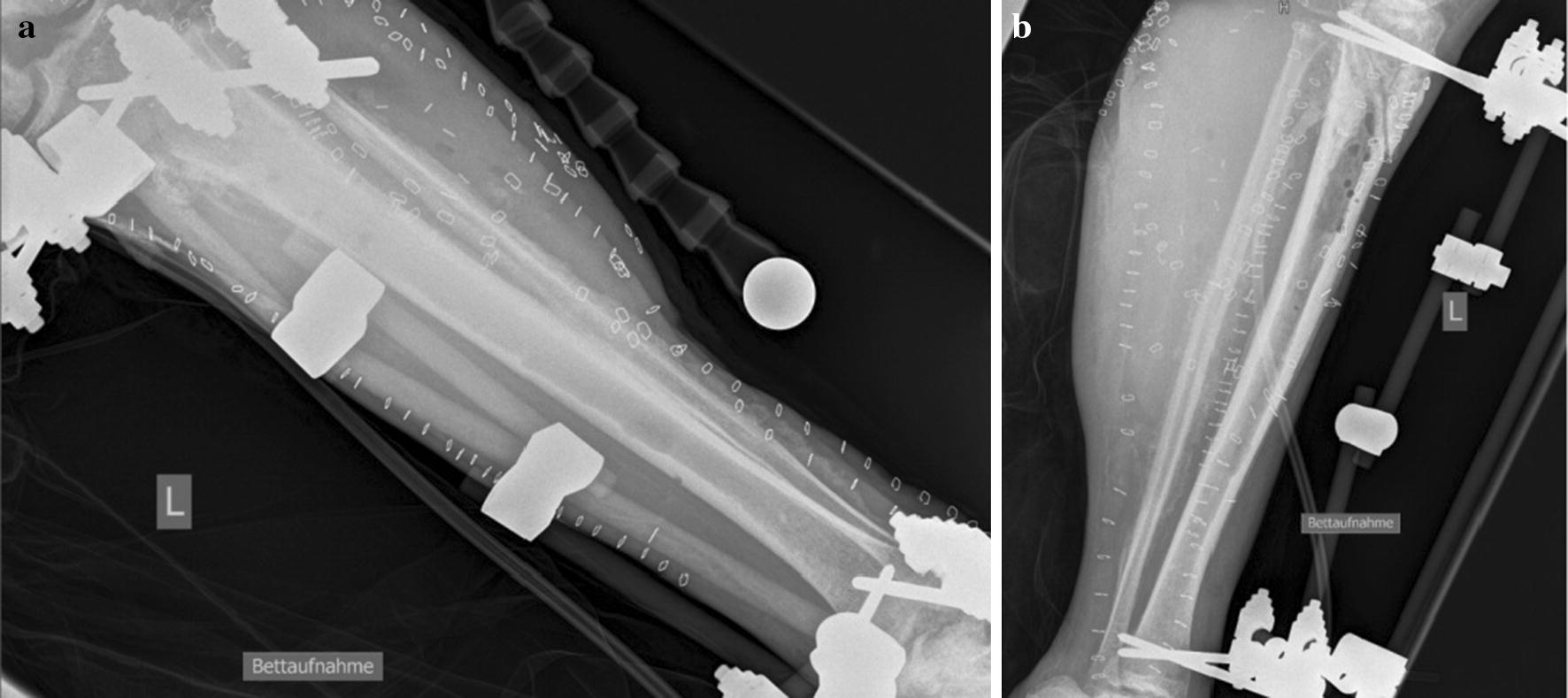
Axial anterior external fixator for securing soft tissue coverage. **a** X-ray a.p. view and **b** X-ray lateral view

**Fig. 7:**
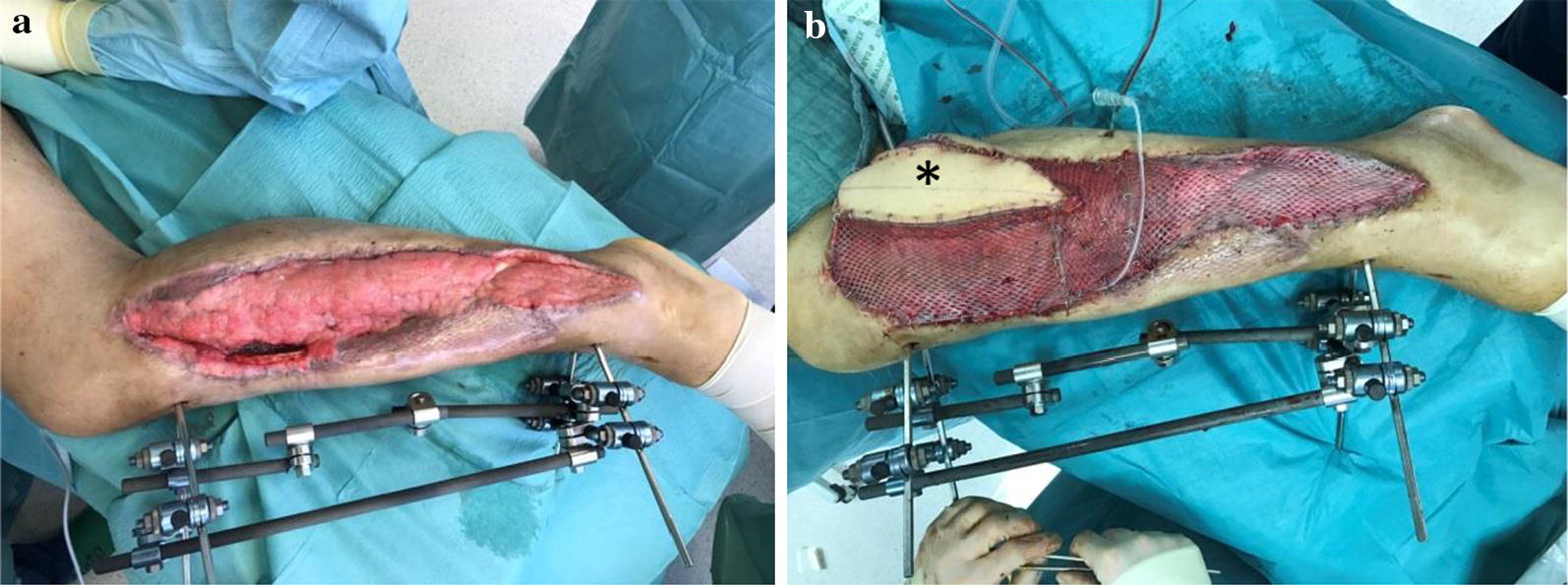
Wound before soft tissue coverage and after full-thickness muscle flap with monitor in situ. **a** Lateral view before soft tissue coverage and **b** lateral view after full-thickness muscle flap with monitor island (*)

**Fig. 8:**
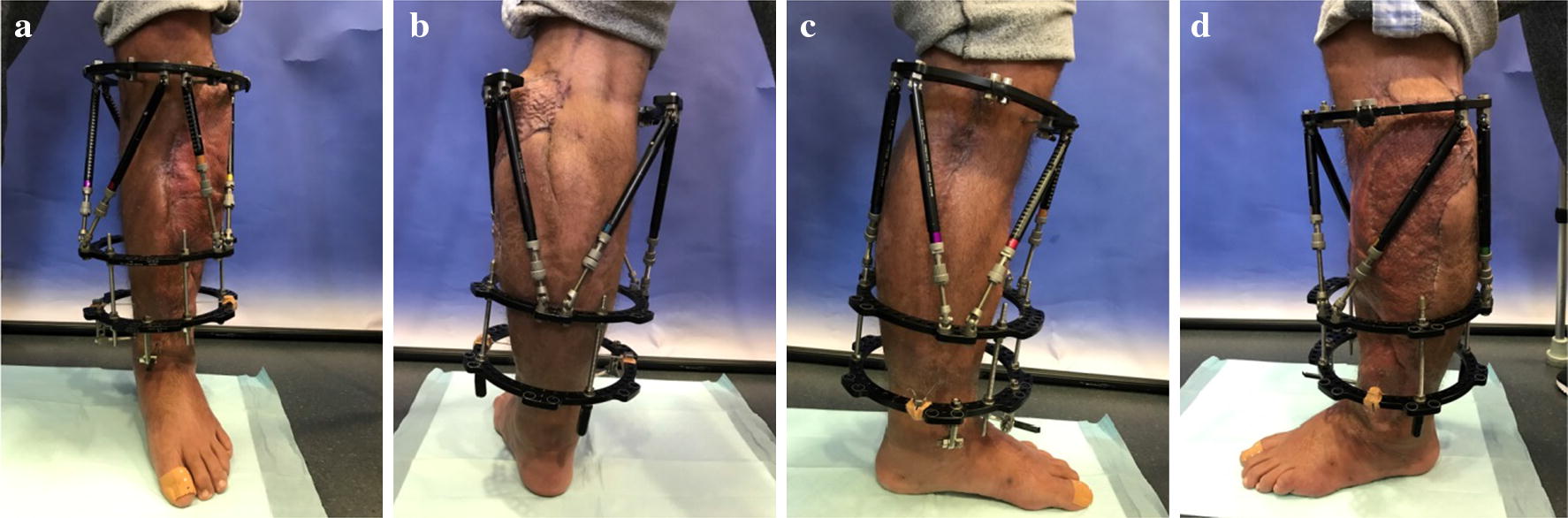
Cosmetics 6 months after trauma with attached TSF fixator. **a** Ventral view, **b** dorsal view, **c** medial view and **d** lateral view

**Fig. 9:**
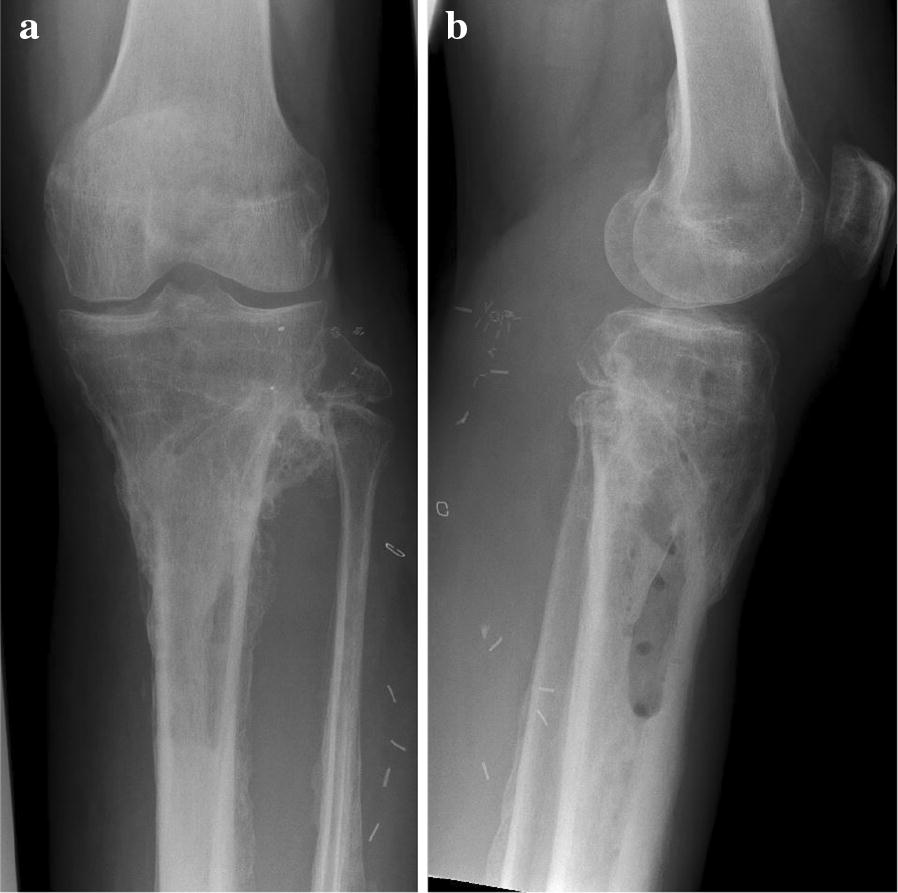
Osseous consolidation 12 months after initial trauma. **a** X-ray a.p. view and **b** X-ray lateral view

Initial surgical management included removal of the osteosynthesis material and necrotic split-thickness graft together with aggressive extensive osseous debridement, resection of necrotic muscles of the anterior lower leg compartment and negative pressure wound therapy. Radical intramedullary debridement was performed using an intramedullary reaming device (SynReam/RIA^®^ DePuy Synthes GmbH, Zuchwil, Switzerland). After two local debridements, Colistin hand-modelled chains and sticks [24 Mio. IE Colistin (Colistin methanesulphonate sodium), InfectoPharm, Heppenheim, Germany + 40 g Palacos R + G^®^, Heraeus Medical, Wehrheim, Germany] were topically inserted intramedullary as well as into the muscle compartments to establish a local high dose of antibiotics. The intramedullary sticks were formed using a thorax drain as a matrix (Argyle™ Trocar Catheter, 24 Ch, Covidien, Dublin, Ireland) and filled with antibiotic-impregnated cement with a cement applicator gun while the chains were made using hand-modelled pads onto an artificial tape (Mersilene tape, Ethicon Endo-Surgery Inc., Somerville, NJ, USA) (Fig. 5). After eight osseous and soft tissue debridements, revision and changing of the local antibiotic spacers and parenteral antibiotic therapy, the wound smears were negative for bacterial growth of *Acinetobacter baumannii* and *Enterobacter cloacae* complex. Two months after initial trauma, an external fixator (AO Fixateur, DePuy Synthes GmbH, Zuchwil, Switzerland) was mounted to protect the planned soft tissue coverage (Fig. 6). The soft tissue defect zone was covered with a full-thickness ipsilateral latissimus dorsi muscle flap and split-thickness grafts by the department of plastic surgery (Fig. 7). The axial anterior external fixator was replaced with a hexapod external fixator together with a foot plate (Taylor Spatial Frame^®^, Smith & Nephew GmbH, Hamburg, Germany) to sequentially address the malalignment of the proximal tibia as well as to perform a gradual correction of the fixed drop foot. No further surgical interventions such as dorsal release or arthrotomy of the ankle joint were performed to reduce the risk for dissemination of the infection. From November 2016 to January 2017, the hexapod fixator was adjusted by the patient with a defined scheme after computed planning for the sequential correction (Fig. 8). Following the completion of treatment (12 months after trauma), the patient showed good functional recovery without neurological or vascular symptoms except the persisting sensomotoric peroneal lesion and is returning to his previous occupation and to low-risk sports activities. ROM was extension/flexion: 0°/0°/120°; VAS was 1 of 10. Plain radiographs showed complete osseous consolidation at final follow-up (Fig. 9).

### Microbiology

Initial local wound smears showed *Acinetobacter baumannii* (Carbapenem resistant) as well as *Enterobacter cloacae* complex. *Enterobacter cloacae* complex was resistant to Ampicillin, Amoxicillin, Piperacillin+/−Tazobactam, Tigecycline, Cefuroxime, Cefotaxime, Ceftriaxone, Gentamycin, Tobramycin, Ciprofloxacin and Moxifloxacin. *Acinetobacter baumannii* was resistant to Fosfomycin, Ampicillin, Amoxicillin, Amoxi-Clavulanate, Ampicillin/Sulbactam, Piperacillin+/−Tazobactam, Tigecycline, Cefuroxime, Cefotaxime, Ceftriaxone, Cefepime, Ceftazidime, Meropenem (MHK > 32), Gentamycin, Tobramycin, Amikacin, Co-trimoxazole, Ciprofloxacin and Moxifloxacin but sensitive to Colistin. Topical application of antibiotics was performed using Colistin cement (24 Mio. IE Colistin (Colistin methanesulphonate sodium, InfectoPharm, Heppenheim, Germany) + 40 g Palacos R + G^®^, Heraeus Medical, Wehrheim, Germany). Systemic parenteral antibiotic treatment was performed using Colistin (1.10.2016 to 30.11.2016; 9 Mio I.E. loading dose followed by 4,5 Mio IE i.v. two times a day), Sulbactam (15.10.2016 to 30.11.2016; 3 g i.v. four times a day) and Fosfomycin (7.10.2016 to 30.11.2016; 5 g i.v. three times a day). After the detection of *Corynebacterium jeikeium* but eradication of *Acinetobacter baumannii* and *Enterobacter cloacae* complex, Vancomycin was added to the systemic antibiotic therapy with a trough level set at 15 mg/l from 4.11.2016 to 30.11.2016. Systemic levels of Vancomycin reached therapeutic levels. The patient suffered no neuro-, oto- or nephrotoxic side effects in the whole follow-up period controlled by neurologic assessment and laboratory chemical kidney retention parameters. Plasma levels of Colistin were not measured.

### Reconstructive/plastic surgery procedure

The large defect located at the anterolateral portion of the left lower leg with a size of approximately 50 × 12 cm and exposed tibial fracture zone required a large microvascular free flap to cover the defect and to support the previously infected wound with a well-vascularized, immunocompetent tissue. Preoperative CT angiography excluded the anterior tibial artery as recipient vessel so that end-to-side anastomoses to the popliteal vessels were planned. Due to the defect size and required pedicle length, the ipsilateral latissimus dorsi muscle was chosen for defect coverage. Time point of surgery was when wound smears were negative and local macroscopic condition showed vital granulation. It was harvested as a musculocutaneous flap with a monitor skin island to allow easy postoperative monitoring of the flap’s perfusion. Intraoperatively, the lateral sural artery, the nutrifying vessel of the lateral gastrocnemius muscle, was found to have a small-enough calibre difference to the thoracodorsal vessels of the latissimus flap’s pedicle to allow end-to-end anastomoses. Anastomoses of the artery and accompanying vein were performed with 9-0 Ethilon single knot sutures with help of a microscope (OPMI Pentero 900^®^, Carl Zeiss AG, Oberkochen, Germany) under 10× magnification. Intravenous injection of indocyanine green (ICG) and fluorescence microscopy showed patency of anastomoses and assured perfusion of the flap. Size of the flap and pedicle length allowed sufficient coverage of the exposed tibia. The flap and the adjacent exposed muscles were covered with 0.3-mm-thick, 1:1.5 meshed split skin grafts harvested from the contralateral thigh. Neither intra- nor postoperative complications occurred. After 1 week, the monitor island was resected, replaced by a skin graft and the mobilization of the patient started.

## Discussion

Open proximal tibial fractures with or without bone loss continue to be challenging for the trauma surgeon [18, 19]. Particularly infected situations involving osseous and soft tissue components dramatically increase the complexity of the situation in which limb salvage is not always predictable. The case at hand presents the combination of six components: radical osseus and soft tissue debridement, intramedullary debridement using the RIA system, application of custom-made cement spacers with high dose of antibiotics combined with systemic antibiotic therapy, flap coverage and hexapod technology to gradually reconstruct both valgus malalignment and drop foot correction without soft tissue release [20–22]. The TSF was used to reconstruct both valgus malalignment and drop foot individually specified for the patient who adjusted the fixator according to a protocol by himself. This can be seen as an advantage towards the traditional Ilizarov fixator. Success of this case is based on the multidisciplinary and individualized approach also in conjunction with microbiologists to achieve the optimal result.

Despite extensive surgery, high dosage of topic antibiotics as well as appropriate parenteral antibiotic therapy is needed to eradicate multi-drug-resistant bacteria and protect soft tissue healing and osseous consolidation. Chen et al. showed in a metaanalysis the effect and importance of Colistin, as well as several other authors [23–25]. Local (topic) and systemic (parenteral) antibiotic therapy in combination with bone cement (PMMA) is used as for infected joints or bone situations [24, 26–29].

However, to the best of our knowledge, this is the first case report in the current literature showing a successful eradication of a multi-drug-resistant *Acinetobacter baumannii* and *Enterobacter cloacae* complex in an open fracture of the proximal tibia combining the demanding procedures described above. Krajewski et al. showed an eradication of an extensively drug-resistant *Pseudomonas aeruginosa* in a case of an open distal femoral fracture using a tobramycin-impregnated PMMA spacer together with Colistin and published the scheme with local beads and spacers impregnated with Colistin we adapted in our patient [30]. In a comparable case, Papagelopoulos et al. eradicated *Pseudomonas aeruginosa* in an infected total knee arthroplasty with implant removal, debridement, Colistin cement spacer, parenteral colistin therapy for 6 weeks and secondary revision arthroplasty [31]. Beieler et al. showed a single eradication of *Acinetobacter baumannii* in a patient with total hip arthroplasty without implant removal and monotherapy of imipenem/cilastatin [32]. Pasticci et al. observed the tolerability and efficacy of long-term treatment in a combination of daptomycin, ceftazidime and colistin showing controllable side effects with special regard to nephro- and neurotoxicity making it eligible for treatment of multi-drug-resistant osseous infections [33].

## Conclusion

Aggressive surgical debridement, topical antibiotics in high dose, complex external fixation and osseous as well as joint correction with a hexapod fixator, individualized systemic parenteral antibiotic therapy and demanding soft tissue reconstruction with a free muscle flap were needed to handle our case of a multi-drug-resistant bacteria-infected open tibial fracture. A multidisciplinary approach with orthopaedic surgeons for debridement, planning and establishing osseous and joint correction and consolidation, plastic surgeons for microvascular muscle flaps for soft tissue defect coverage as well as clinical microbiologists for the optimized anti-infective treatment is essential in these challenging rare cases.
